# Transplant of microbiota from Crohn’s disease patients to germ-free mice results in colitis

**DOI:** 10.1080/19490976.2024.2333483

**Published:** 2024-03-27

**Authors:** Irshad Ali Sheikh, Jared Bianchi-Smak, Daniel Laubitz, Gabriele Schiro, Monica T. Midura-Kiela, David G. Besselsen, Gayatri Vedantam, Sara Jarmakiewicz, Rafał Filip, Fayez K. Ghishan, Nan Gao, Pawel R. Kiela

**Affiliations:** a Daniel Cracchiolo Institute for Pediatric Autoimmune Disease Research, Steele Children’s Research Center, Department of Pediatrics, University of Arizona, Tucson, AZ, USA; b Department of Biological Sciences, Rutgers University, Newark, NJ, USA; c Pediatrics, University Animal Care, University of Arizona, Tucson, AZ, USA; d School of Animal and Comparative Biomedical Sciences, The University of Arizona, Tucson, AZ, USA; e Institute of Health Sciences, Medical College of Rzeszow, Rzeszow University, Rzeszow, Poland; f Institute of Medicine, Medical College of Rzeszow University, Rzeszow, Poland; g Department of Gastroenterology with IBD Unit, Clinical Hospital, Rzeszow, Poland; h Department of Immunobiology, University of Arizona, Tucson, AZ, USA

**Keywords:** Crohn’s ileocolitis, microbiota, fecal microbiome transplant, germ-free mice, inflammation

## Abstract

Although the role of the intestinal microbiota in the pathogenesis of inflammatory bowel disease (IBD) is beyond debate, attempts to verify the causative role of IBD-associated dysbiosis have been limited to reports of promoting the disease in genetically susceptible mice or in chemically induced colitis. We aimed to further test the host response to fecal microbiome transplantation (FMT) from Crohn’s disease patients on mucosal homeostasis in ex-germ-free (xGF) mice. We characterized and transferred fecal microbiota from healthy patients and patients with defined Crohn’s ileocolitis (CD_L3) to germ-free mice and analyzed the resulting microbial and mucosal homeostasis by 16S profiling, shotgun metagenomics, histology, immunofluorescence (IF) and RNAseq analysis. We observed a markedly reduced engraftment of CD_L3 microbiome compared to healthy control microbiota. FMT from CD_L3 patients did not lead to ileitis but resulted in colitis with features consistent with CD: a discontinued pattern of colitis, more proximal colonic localization, enlarged isolated lymphoid follicles and/or tertiary lymphoid organ neogenesis, and a transcriptomic pattern consistent with epithelial reprograming and promotion of the Paneth cell-like signature in the proximal colon and immune dysregulation characteristic of CD. The observed inflammatory response was associated with persistently increased abundance of *Ruminococcus gnavus, Erysipelatoclostridium ramosum, Faecalimonas umbilicate, Blautia hominis*, *Clostridium butyricum*, and *C. paraputrificum* and unexpected growth of toxigenic *C. difficile*, which was below the detection level in the community used for inoculation. Our study provides the first evidence that the transfer of a dysbiotic community from CD patients can lead to spontaneous inflammatory changes in the colon of xGF mice and identifies a signature microbial community capable of promoting colonization of pathogenic and conditionally pathogenic bacteria.

## Introduction

Inflammatory bowel diseases (IBDs), which include Crohn’s disease (CD) and ulcerative colitis (UC), are chronic remitting/relapsing inflammatory disorders of the gut. There is a prevailing consensus that at the root of the IBD etiology is an inappropriate immune response to the components of the commensal gut microbiota in a genetically susceptible host that leads to persistent inflammation and tissue damage^1^. Although the existence of a heritable component of IBD is beyond doubt, with approximately 240 loci statistically associated with the risk of developing IBD,^2,3^ genetics alone cannot explain the rising incidence of IBD in recent decades, rising prevalence of early and very early onset IBD, or an increasing risk of IBD in individuals migrating from low- to high-incidence geographic regions.^4^ Thus, environmental, dietary, and microbial factors are believed to be strong components of IBD susceptibility. Several lines of evidence support the role of luminal constituents in promoting IBD risks. Clinically, CD, UC, and pouchitis affect intestinal segments with the highest bacterial concentrations. All forms of IBD are associated with altered composition and metabolic activity of the resident microbiota and increased abundance of mucosal-associated bacteria, including enteroinvasive species. Antibiotics remain a part of a clinician’s arsenal of treatment options to decrease postoperative or septic complications in IBD patients and have been used as adjuvant treatment for fulminant colitis.^5^ Mucosal inflammation in Crohn’s ileitis can be improved by fecal flow diversion by loop ileostomy and reactivated following reinfusion of ileostomy contents.^6^ However, clinical observations alone do not prove causality. Animal studies, especially with germ-free (GF) mice, have provided a valuable tool to test the relationship between the gut microbiota and host inflammatory response while maintaining control of the host genotype, diet and other environmental influences. Several studies have demonstrated the requirement for gut commensals for the development of spontaneous colitis in genetically susceptible mice^6–10^

Considering that altered gut microbial community composition and metabolic activity have been well documented in both UC and CD patients,^1,11^ several investigations have tested the effects of the transfer of complex/complete human fecal or mucosa-associated microbiota from healthy donors and donors with IBD on the development of intestinal inflammation in mice^12–15^ This body of work conclusively demonstrated that the IBD-associated microbiota promotes inflammatory responses in genetically susceptible hosts (IL10^−/−^), during chemical challenge with dextran sulfate sodium (DSS), or in an adoptive T-cell transfer model of colitis. However, despite subtle changes in inflammatory tone, promotion of mucosal Th2 and RORγt^+^ T helper cells (Th), and reduced induction of FoxP3^+^RORγt^+^ regulatory T cells (Tregs),^13^ no overt mucosal inflammation or damage was noted in wild-type or untreated IBD-humanized mice. These studies suggest that the IBD-associated microbiome promotes inflammatory responses in experimental colitis but is insufficient to independently induce mucosal inflammation. Interpretation of these studies is, however, more complicated due to variable engraftment of human microbiota in GF mice, the inherent but difficult to control losses of potential pathobionts, and inconsistent changes in the gut microbiota composition in IBD patients in general. As an example, the composition of fecal microbiota from IBD patients in Britton et al.^13^ was indistinguishable from healthy donors in beta-diversity analysis.

Here, we examined the impact of transferring fecal microbiota from patients with Crohn’s ileocolitis to wild-type GF mice. We demonstrated that fecal microbiome transplantation (FMT) from these CD donors was sufficient to induce spontaneous colitis but not ileitis in wild-type murine recipients. The observed changes were consistent with chronic Crohn’s-like colitis as determined by morphological, immunohistochemical, and transcriptomic analyses. Our results demonstrate for the first time that microbiota from IBD patients is sufficient to induce colonic pathology in the absence of underlying genetic susceptibility or chemical stimuli.

## Materials & methods

### Fecal samples

Human fecal samples were collected by Dr. Rafal Filip at the Center for Comprehensive Treatment of Inflammatory Bowel Disease Regional Hospital No. 2 in Rzeszow, Department of Internal Diseases, University of Rzeszow, Poland, under approval by the local bioethics commission (approval number KE-0254/68/2015). Samples were collected from 29 healthy control patients and 35 Crohn’s disease (CD) patients. Written informed consent forms were obtained from all study participants. Patients with clear histological and endoscopic features of CD who responded to anti-TNF therapy (infliximab or adalimumab) in early phase of maintenance treatment (less then 3 months), were included. CD and healthy control subjects were not treated with any antibiotics for at least 5 months before stool collection and had negative tests for intestinal bacterial infections such as *Clostridium difficile*, or other infections such as hepatitis B, hepatitis C, or HIV (human immunodeficiency virus). Exclusion criteria included the presence of ostomy, known intrabdominal fissures and abscesses, and active perianal disease. Collected stool samples were stored at −80°C until use. Patient characteristics are listed in Supplemental Table S1. Deidentified specimens were transferred to the Kiela lab at the University of Arizona. DNA was extracted from stool samples with a QIAamp PowerFecal Pro DNA Kit (QIAGEN, Hilden, Germany) and analyzed by 16S amplicon profiling. Based on the genus-level analysis, we selected 5 samples from healthy controls (HC) and 6 samples from patients with Crohn’s ileocolitis (Montreal classification L3; CD_L3) for association in GF mice. Samples were selected to reflect the consensus in taxonomic analysis of CD fecal samples reported in peer-reviewed literature. Equal weights of HC and CD_L3 samples were pooled, and suspensions were prepared with reduced sterile PBS on day 0. Glycerol stocks were stored at −80°C and used for the subsequent gavages on days 7 and 14.

### Mice

Animals used in the experiments were handled in accordance with the University of Arizona University Animal Care (UAC) guidelines and with an approved IACUC protocol (07–126; Kiela). 9-week-old germ-free male and female C57BL/6 mice were transferred to IsoCage P – Bioexclusion cages (Tecniplast) and administered the pooled inoculum in three weekly doses (on days 0, 7, 14) through oral gavage. 9 mice received FMT from healthy controls (HC; 5 females and 4 males) and 13 mice received FMT from patients with Crohn’s ileocolitis (CD_L3; 7 females and 6 males). Body weight was monitored weekly, and mice were euthanized on day 21.

### Histopathology

Tissue sections were excised from the ileum, proximal and distal colon, fixed in 10% buffered formalin, transferred to 70% ethanol, and embedded in paraffin. Samples were cut into 5-μm-thick sections, and hematoxylin and eosin (H&E) staining was performed using the standard procedure. Pathology scoring was performed blinded to the experimental design and group assignment. Scoring was based on lesion scoring criteria for mouse intestinal lesions modified from Burich et al.^16^ Criteria included mucosal proliferation, inflammation, extent of proliferation, and extent of dysplasia. The criteria range from total scores of 0 (no colitis) to 13 (severe colitis with ulcerations).

### Immunohistochemistry and immunofluorescence

The methods for immunofluorescence (IF) are detailed in Yu et al.^17^ Briefly, tissue sections were cut to a thickness of 5 µm, rehydrated, and subjected to antigen retrieval with citrate acid buffer (pH 6). Slides were then incubated in blocking buffer at room temperature for 1 hour (1X PBS with 0.1% Triton X-100, 2% normal serum, 2% BSA). Slides were probed with primary antibodies and incubated at 4°C overnight. The primary antibodies used were sheep anti-CD74 (R & D, AF7478), rabbit anti-Lysozyme (BioGenex, AR024), mouse anti-E-cadherin (BD Biosciences 610,404), rabbit anti-Mptx2 (Abcam, ab238123), rabbit anti-MMP7 (Cell Signaling D4H5, 3801), rabbit anti-NF-κB p65 (Cell Signaling, D14E12, 8242), rabbit anti-CD3 (Abcam, ab16669, SP7), and rabbit anti-CD14 (Novus Biological, NBP2–67630). The following day, slides were incubated with Alexa Fluor-conjugated fluorescent secondary antibodies and DAPI for 1 hour at room temperature. Slides were mounted with Prolong Gold Antifade Mountant (Invitrogen, P36930). Images were collected using a Nikon Eclipse TE2000-U for brightfield or epifluorescence. Confocal images were collected with a Zeiss LSM980. Images were analyzed with ImageJ, NIS-Elements, or Zen software.

### Microbiota analysis

#### 16S amplicon profiling, shotgun metagenomics and bioinformatics

The hypervariable V4 region of the 16S rRNA gene was amplified from each sample with unique barcoded reverse primer (806 R) and forward primer (515F) using MyFi™ Mix (Bioline Meridian, Cat No. BIO-25050). Both reverse and forward primers were extended with sequencing primer pads, linkers, and Illumina adapters.^18^ PCR was performed using a LightCycler 96 (Roche) in a final volume of 40 μL. Amplicons were quantified using a Quant-It PicoGreen dsDNA Assay kit (Thermo Fisher Scientific, Cat No. P7589) according to the manufacturer’s protocol. Equal amounts of 240 ng of amplified DNA from each sample were pooled and cleaned using an UltraClean PCR Clean-Up Kit (QIAquick PCR Purification Kit, Qiagen, Cat No. 28104). Pooled amplicons were diluted and denatured with 0.2 N NaOH. The library was sequenced at the University of Arizona PANDA CORE for Genomics and Microbiome Research using the MiSeq platform (Illumina) and custom sequencing primers.^18^ Due to the limited sequence diversity among 16S rRNA amplicons, 5% of the PhiX Sequencing Control V3 (Illumina, Cat No. FC-110-3001) made from phiX174 was used to spike the library and increase the diversity.

The raw sequencing data were demultiplexed using the *idemp* script (https://github.com/yhwu/idemp). Filtering, dereplication, chimera identification, and merging of paired-end reads were performed with dada2.^19^ The amplicon sequence variant (ASV) taxonomy was assigned using the Ribosomal Database Project (RDP) classifier^20^ against SILVA database release 138.^21^ Taxonomic richness and diversity (Shannon and Simpson indices) were calculated on rarefied data (depth: human 21,546, mice 13,856) and statistically analyzed using the Kruskal‒Wallis rank sum test followed by Dunn’s multiple-comparison test with Bonferroni correction. The contribution of different metadata variables to community composition was evaluated with permutational multivariate analysis of variance (PERMANOVA) on Bray‒Curtis dissimilarities calculated on rarefied data and plotted onto a nonmetric multidimensional scaling (NMDS) ordination. All analyses were performed in R (ver 4.2.1)^22^ aided by the package ggplot2 for data visualization.^23^ Differential abundance analysis was performed at the ASV level using DEseq2.^24^

### Shotgun metagenomics bioinformatic approaches

Library preparation for shotgun metagenomics was performed using a DNA prep Tagmentation kit (Illumina, San Diego, CA, USA). The quality and quantity libraries were determined with a 4150 TapeStation DNA bioanalyzer (Agilent Technologies, Palo Alto, CA, USA). All samples were shotgun sequenced on a 2 × 150 bp NextSeq550 platform (Illumina, San Diego, CA, USA) at the University of Arizona PANDA CORE for Genomics and Microbiome Research.

Short (<50 bp) and low-quality reads were removed with trimmomatic V7.3.0.^25^ Human contamination was removed by mapping the reads to the human reference genome (Build 38) using bowtie2 V2.3.5.1.^26^ A total of 30,844,794 to 89,070,217 read pairs per sample remained after quality and host sequence trimming. Paired-end reads were taxonomically profiled using MetaPhlAn3 v 3.0.14,^27^ with standard settings and the CHOCOPhlAn v30 database. The results were then manually screened for potential known pathogens. De novo assembly was performed using megahit v1.2.9,^28^ and contigs shorter than 500 bp were discarded. Prodigal V2.6.3^29^ was used to predict protein-coding sequences. Functions were inferred by comparison to the Kyoto Encyclopedia of Genes and Genomes (KEGG) database using Kofam scan version 1.3.0.^30^ Coverage was evaluated by Contigs containing the KEGG orthology K11063 (TcdA and TcdB from *C. difficile*) were extracted and further annotated using Prokka 1.14.6^31^ supplemented with PFAM HMM database v. 35.^32^ and manually curated through blastx alignments.^33,34^ Full-length contigs were also blasted in the full NCBInt database^35^ (downloaded September 2022) using a standalone blast algorithm.

### C. difficile *qPCR analysis*

DNA isolated from fecal samples of recipient mice was used for TaqMan qPCR analysis of toxigenic *C. difficile* 16S rRNA and *tcdA* and *tcdA* toxin genes according to a method by Kubota et al.^36^ using a LightCycler 96 System (Roche) and the primers and probes listed in Supplemental Table S2.

### RNA sequencing and analysis

Total RNA was isolated from the ileum, proximal and distal colon using the AllPrep DNA/RNA Mini Kit (Qiagen). The RNA quality was verified using Tape Station 4150 (Agilent), and the RNA quantity was verified using Qubit RNA High Sensitivity reagent (Thermo Fisher). The libraries for sequencing were prepared using Illumina Stranded Total RNA Prep, Ligation with Ribo-Zero Plus kit with 100 ng of RNA as input. The quality of the final libraries was tested on a TapeStation 4150 (Agilent) and quantified with PicoGreen reagent (ThermoFisher). All libraries were diluted to a final concentration of 4 nM and combined into an equimolar pooled library prior to sequencing. The pooled library was diluted, denatured, and sequenced on a NextSeq 550 with high-output chemistry. FASTQ files were imported into the Partek Flow software suite (Partek, Inc., Chesterfield, MO), and 3’ base trimming was performed to remove reads with Phred quality scores below 20. Spliced Transcripts Alignment to a Reference (STAR)^37^ was used to align the trimmed reads to the mm39 mouse genome assembly and annotated with the current RefSeq database. Median ratio normalization was performed, and differential gene expression was analyzed with DESeq2.^24^ Principal component analysis (PCA) was performed for each gut segment to reduce dimensionality and visualize the differences in gene expression profiles. Gene set enrichment analysis (GSEA) was performed with GSEA v4.3.2 software (a joint project of UC San Diego and Broad Institute).^38,39^

### Quantitative RT-PCR

200 ng of total RNA was used for reverse-transcription using the Universal Transcriptor cDNA synthesis kit (Roche). Real-time qPCR was performed on these samples to validate expression of selected genes identified in RNAseq analysis (genes (TNFα, CD74, CD14, Cxcl9, Cxcl10, and CIITA) using LightCycler 96 Thermocycler (Roche). Cq values were obtained using LightCycler 96 software (version 1.1.0.1320) and were analyzed using the comparative Ct method as the means of relative quantification, normalized to TATA box binding protein mRNA (TBP) as the housekeeping gene and relative to a calibrator (normalized Ct value obtained from mice colonized with HC microbiota) and expressed as 2^−^ΔΔ^Ct^ (Applied Biosystems, FosterCity, CA; UserBulletin #2: RevB “Relative Quantification of Gene Expression”).

## Results

### Characterization of human CD microbiota

**Figure 1.**
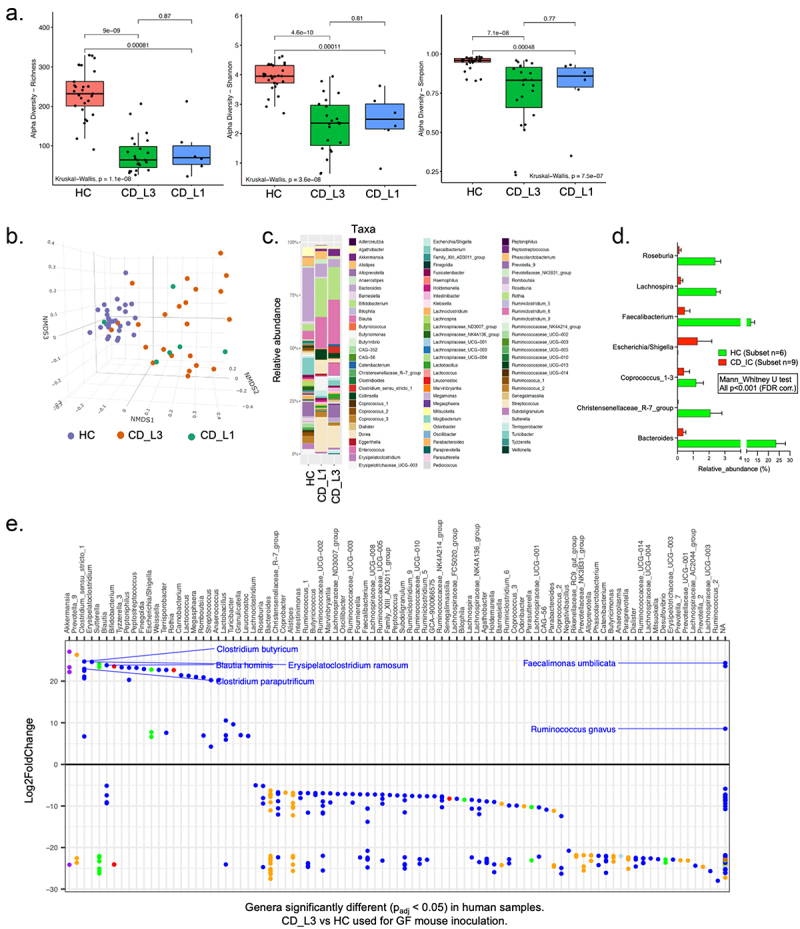
Analysis of fecal microbiota from healthy and CD patients and selected samples for mouse colonization.

The microbiota of diagnosed CD patients was characterized prior to administration to GF mice. Patient characteristics, including stratification based on the Montreal classification of IBD^40^ are listed in Supplemental Table S1. Fecal samples were collected from each participant and sequenced using 16S amplicon profiling. Consistent with prior literature, species richness, Shannon index, and Simpson index used as measures of alpha diversity were all significantly lower in CD_L1 and CD_L3 compared to HC (Figure 1a). Beta diversity was visualized using nonmetric multidimensional scaling (NMDS) for the three groups. We observed close clustering of the HC samples. Consistent with the reported heterogeneity of microbial composition in IBD patients, the distribution of CD_L1 and CD_L3 samples was less consistent but with minimal overlap with HC samples (Figure 1b). Figure 1c and Supplemental Fig. S1 depict a visual representation of the relative genus abundance in each of the three groups (only genera with a relative abundance of >0.1% are shown).

(a) Alpha diversity indices of the fecal microbiome from healthy controls (HCs) and Crohn’s disease patients with ileitis (CD_L1) and ileocolitis (CD_L3). (b) Beta diversity (nonmetric multidimensional scaling; NMDS) analysis of HC, CD_L1 and CD_L3 fecal samples. (c) Compositional depiction of the most abundant genera in HC, CD_L1 and CD_L3 fecal samples. (d) Relative abundance of selected genera used for selection of samples for inoculation of GF mice. (e) Comprehensive analysis of differentially abundant ASVs in samples selected for GF mouse inoculation using DESeq2.

For GF mouse inoculation, microbial communities were generated by pooling samples from the patient groups. Samples were not chosen randomly but rather to approximate the microbiome composition of typical Crohn’s ileocolitis based on the consensus data in published literature, with a focus on seven genera, *Roseburia, Lachnospira, Faecalibacterium, Escherichia/Shigella, Coprococcus_1–3, Christensenellaceae_R-7_group, and Bacteroides*. Figure 1d shows those genera in samples selected for pooling an inoculation of GF mice. DESeq2 analysis of the selected samples shown in Figure 1e provides a more comprehensive analysis of the bacterial composition of samples from HC and CD_L3 selected for pooling and inoculation.

To test whether the selected samples contained known pathogens that could influence the resulting phenotype in recipient GF mice, pooled inocula from HC and CD_L3 patients as well as stool samples collected from recipient mice at the time of euthanasia (pooled separately from males and females) were analyzed by shotgun metagenomics. Of the 23 viruses or phages that were detected in human inocula, none were detected in colonized mice. *Saccharomyces cerevisiae* was detected in CD_L3 (relative abundance of 0.43%) but not in HC inocula and was not detected in recipient mice. Of the 309 bacterial species detected in human samples, only 72 were effectively engrafted in GF mice (23.3%) (Supplemental Table S3). Engraftment was higher in the recipients of the HC microbiota (27.9%) than in those of the CD_L3 microbiota (8.6%). Of the 72 engrafted species (present in both donor samples and in mouse recipients), none represented a known human or murine pathogen (Supplemental Tables S3 and S4).

### Characterization of microbiota in humanized xGF mice

Although the transferred microbiota did not include known pathogenic species, we identified three species that were entirely absent or below the detection limit in human inocula (HC or CD_L3) but bloomed exclusively in xGF mice colonized with CD_L3. These were *Eisenbergiella massiliensis* (to 1.64% in female mouse recipients and 3.55% in male recipients), *Clostridium_sp_7_2_43FAA* (to 0.12% in females and 0.01% in male recipients), and *Clostridioides difficile* (to 4.74% in females and 3.97% in male recipients) (Supplemental Table S4). Further metagenomic analysis identified two contigs containing a complete *C. difficile* pathogenicity locus and having high sequence similarity with *C. difficile* strains Cd10 and Cd11 (NCBI CP037827.1 and CP037822.1, Supplemental Fig. S2). We further confirmed this observation using qPCR (Supplemental Fig. S3). Each mouse that received the CD_L3 pooled microbiome was positive for a toxigenic strain of *C. difficile*, as indicated by the presence of *C. difficile* 16S DNA and the tcdA and tcdB genes (Supplemental Fig. S2 and S3).

**Figure 2.**
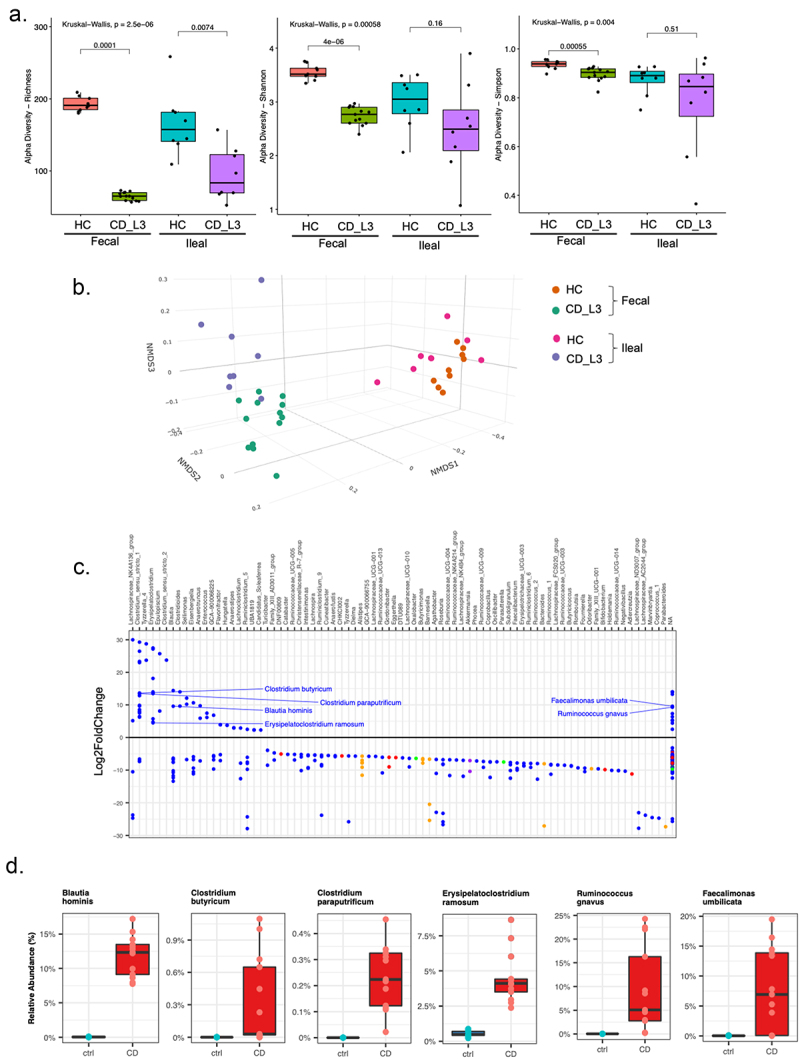
Analysis of the established microbiomes in GF mice humanized with HC and CD_L3 fecal samples.

The species diversity of both ileal contents and feces of the humanized mice was characterized using 16S sequencing from samples collected from individual mice at the time of euthanasia. Fecal samples of the recipient mice reflected the differences in community diversity among the human specimens, with richness, Shannon, and Simpson indices significantly lower in CD_L3 recipients (Figure 2a). For ileal contents, only richness reflected a significant difference in alpha diversity (Figure 2a). NMDS analysis of beta diversity showed very distinct clustering of samples from recipients of HC vs CD_L2 microbiota and less dramatic but discernible separation of ileal and fecal samples (Figure 2b). Figure 2c depicts the results of DESeq2 analysis of ASVs in fecal samples from humanized mice (ASVs at *p* < 0.5 in CD_L3 vs. HC recipients). The relative abundance of selected species identified as having 100% 16S identity with a given ASV is shown in Figure 2d, including *Ruminococcus gnavus*, a species known for its association with CD^41–45^ whose abundance was significantly increased in CD_L3 donors and xGF recipients.

(a) Alpha diversity indices of fecal and ileal microbiomes from xGF mice humanized with microbiota from HC and CD_L3 patients. (b) Beta diversity (NMDS) analysis of fecal and ileal microbiomes from xGF mice humanized with microbiota from HC and CD_L3 patients. (c) DESeq2 analysis of differentially abundant ASVs in fecal samples from HC and CD_L3 humanized mice. (d) Relative abundance of the six most abundant genera established in the fecal samples of HC and CD_L3 humanized mice.

### Ileum is not impacted by inoculation of CD-associated fecal microbiota

Fig. S4A depicts unaltered villi and crypt structure in the ileum of xGF mice colonized with HC and CD_L3 microbiota, with no histological evidence of ileitis. Ileal lysozyme, an antimicrobial peptide secreted by Paneth cells in response to pathogens^46–48^ was not altered between the groups (Fig. S4B). Similarly, no differences were observed in immunostaining for MMP7, an antimicrobial peptide essential for activation of mouse alpha-defensins^49–51^ (Fig. S4C), or for MPTX2, a Paneth cell-specific mucosal pentraxin^52^ (Fig. S4D). A PCA plot comparing the transcriptomic profiles of the ileum of HC and CD_L3 recipients indicated no distinct clustering (Fig. S4E) and confirmed the immunofluorescence (IF) results for *Lyz1, Mmp7* and *Mptx2* at the mRNA level (Fig. S4F). DESeq2 analysis indicated minimal changes in the gene transcription profile, with the vast majority of altered mRNA being either noninformative or noncoding (Fig. S4G). These observations were confirmed by unbiased histopathology scoring, which did not identify inflammatory changes in the ileum of mice receiving microbiota from CD_L3 patients (Fig. S4H).

### CD microbiota in xGF mice induces colonic inflammation

**Figure 3.**
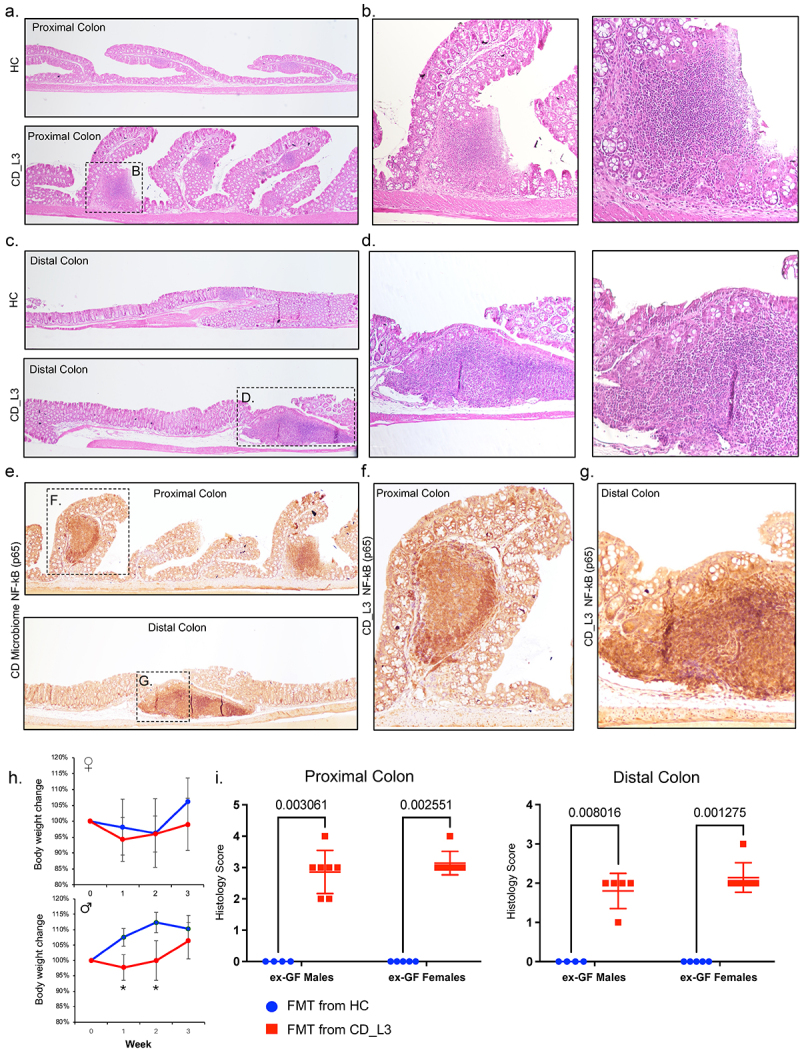
Histological features of mild to moderate colitis in xGF mice humanized with HC and CD_L3 fecal microbiota.

There was no evidence of inflammation in the proximal or distal colon of mice colonized with the HC microbiota (Figure 3a,c). However, in mice colonized with CD_L3 microbiota, both segments showed evidence of mild to moderate colitis, as indicated by mild to moderate hyperplasia, edema, mild immune cell infiltration, enlarged isolated lymphoid follicles and/or neogenesis of mucosal tertiary lymphoid organs (TLO), typical features of chronic colitis in humans (primarily CD) and mice ^53–55^ (Figure 3a–d). Higher magnification showed a loss of regular crypt structure as well as damage or loss of the limiting outer epithelial layer surrounding the TLOs (Figures 3b & 3d). Inflammation was discontinuous, with no case where the inflammation exceeded the length limits of 50% of the analyzed segment.

Representative H&E-stained images of the proximal (a-b) and distal (c-d) colon of humanized mice. (e-g) Expression of the p65 subunit of the NFκB complex in the mucosa of the proximal and distal colon in HC and CD_L3 humanized mice. (h) Relative body weight change in xGF mice. (i) Unbiased histological pathology scoring of the proximal and distal colons of xGF mice humanized with HC and CD_L3 fecal microbiota.

Immunohistochemical (IHC) staining of NF-κB (p65), an established upstream regulator of inflammatory responses, apoptosis, and cytokine signaling, showed high p65 expression within the TLOs of CD_L3 mice, as well as at sites with evident epithelial damage in both segments of the colon (Figure 3e–g). p65 expression in undamaged or uninflamed regions was comparable in both the HC and CD_L3 mice (not shown). None of the experimental mice had symptoms of diarrhea, rectal prolapse, or rectal bleeding, and we did not observe a significant effect of the CD_L3 FMT on relative body weight change in females, and only a very modest and transient delay on weight gain in males (Figure 3h). Pathology scoring blinded to the experimental design and group assignment confirmed the histological/IHC observations in HC and CD_L3 microbiota recipients, with elevated histological scores in the latter group, regardless of the recipient muse sex (Figure 3i).

### Inflammatory signatures in the proximal and distal colon transcriptomes of CD microbiota recipients

**Figure 4.**
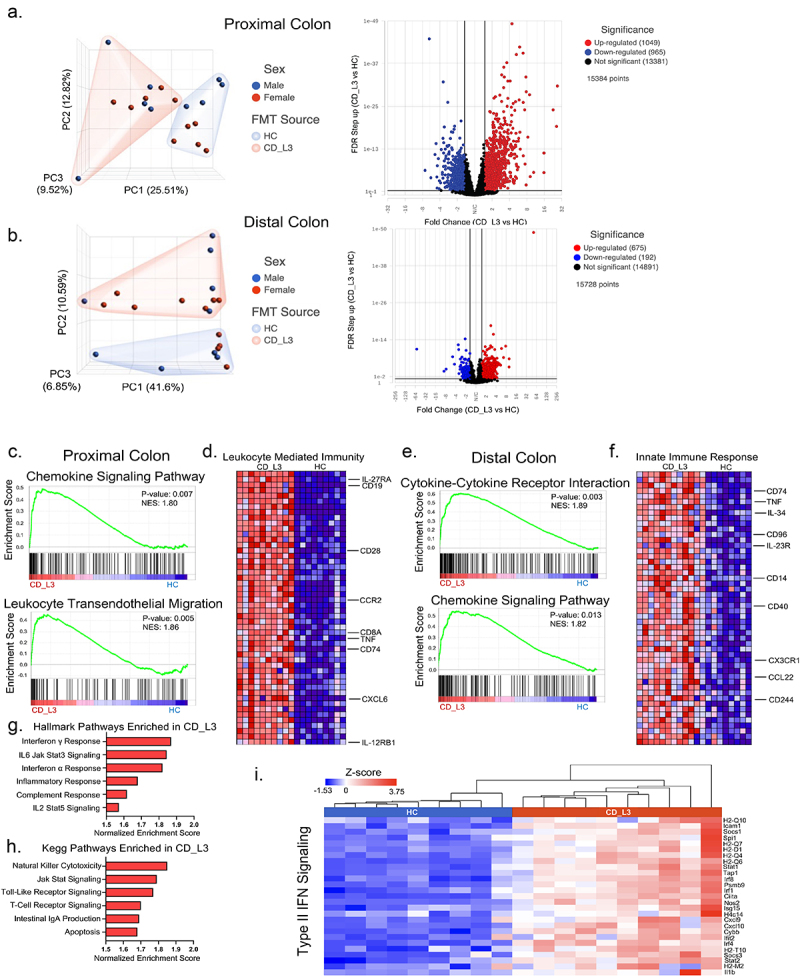
Inflammatory signature of the colonic transcriptome profiling of xGF mice humanized with HC and CD_L3 fecal microbiota.

**Figure 5.**
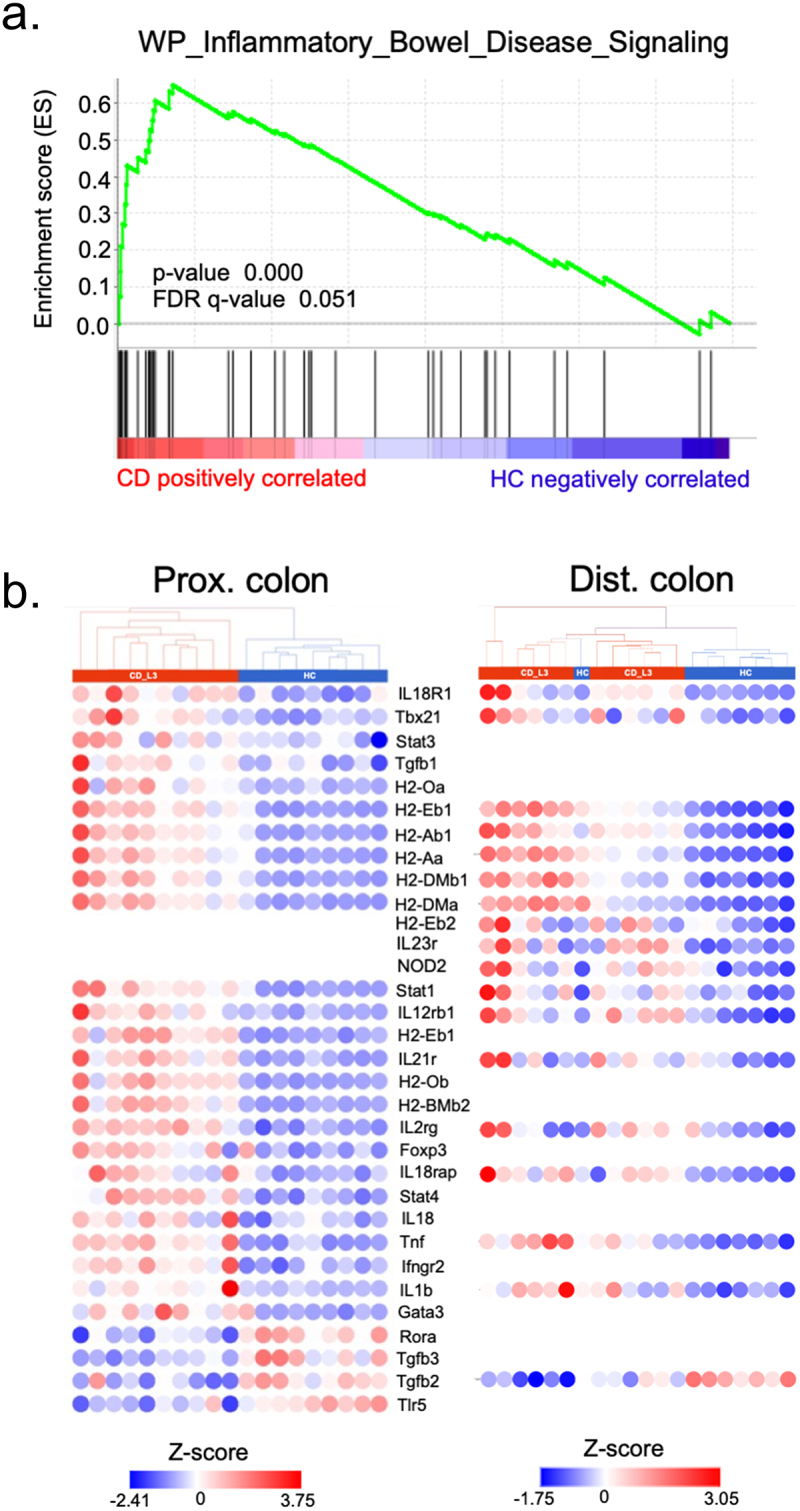
The colonic transcriptomic profile of CD_L3 recipients shows an enrichment in the IBD pathway.

**Figure 6.**
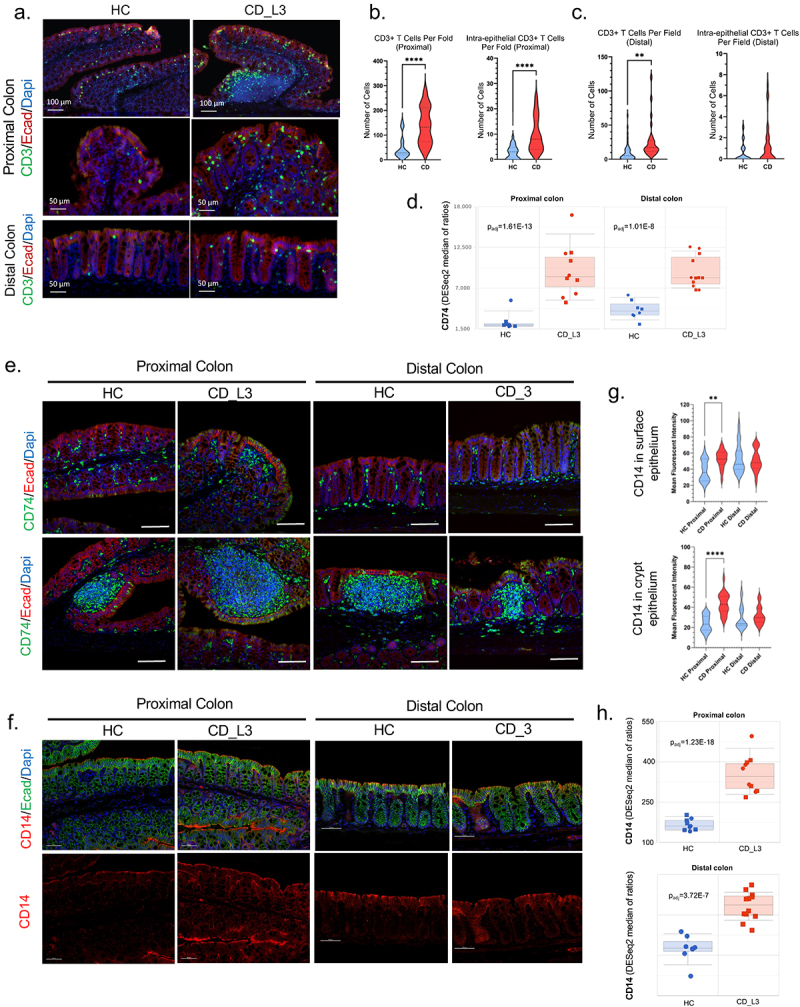
Increased T-cell infiltration and increased expression of epithelial CD74 and CD14 in the inflamed colonic mucosa of xGF mice humanized with HC and CD_L3 fecal microbiota.

Principal component analysis of the proximal and distal colon transcriptomic profiling, with distinct clustering of samples from HC and CD_L3-associated mice, along with accompanying volcano plots are shown in Figures 4a, b, respectively. GSEA with gene sets from the KEGG database was used to identify some of the significantly enriched inflammatory pathways. The leading-edge plots shown in Figure 4c (chemokine signaling and leukocyte transmigration) are consistent with the increased immune cell recruitment shown in Figure 6. We observed enrichment of the GO:BP pathway leukocyte-mediated immunity, with increased expression of TNF, IL-27RA, and CD74 as well as other immune cell-related genes and cytokine receptors (Figure 4d). In the distal colon of CD_L3 recipients, we observed an enrichment in cytokine and chemokine signaling pathways and general upregulation of innate immune signaling-associated transcripts (Figure 4e–f). Other pathways from the MSigDB Hallmark and KEGG pathway collections that were highly enriched in the proximal colon of CD_L3 recipients, along with a breakdown of genes involved in the IFNγ response, are summarized in Figure 4g–i. Importantly, the colonic transcriptomic profile of CD_L3 recipients showed an enrichment in the inflammatory bowel disease signaling pathway (P_adj_ = 0.05), with elevated expression more prevalent in the proximal colonic segment, consistent with independent observations pointing to a more proximal disease (Figure 5a,b). When analyzed in the context of the KEGG IBD pathway (hsa05321), our data point to a picture more consistent with CD than with UC (Fig. S6).

(a-b) PCA and volcano plots demonstrating the extent of differential gene expression in mice colonized with HC and CD_L3 microbiota. (c) GSEA of the chemokine signaling pathway and leukocyte transendothelial migration in the proximal colon. (d) Heatmap of gene expression data of leukocyte-mediated immunity KEGG pathways in the proximal colon. (e) GSEA of cytokine‒cytokine receptor interactions and chemokine signaling pathways in the distal colon. (f) Heatmap of gene expression data of the innate immune response KEGG pathway in the distal colon. (g-h) Other hallmark and KEGG pathways significantly enriched in the proximal colonic transcriptome of mice humanized with the CD_L3 microbiota. (i) Heatmap of gene expression data of the type II interferon signaling pathway in the proximal colon.

(A) GSEA of the proximal colonic transcriptome of humanized mice. (B) Hierarchical clustering and heatmap of genes associated with the IBD pathway differentially expressed in the proximal and distal colon of HC and CD_L3 recipients (with P_adj_<0.05 without fold-change filtering).

### Colitis in CD microbiota recipients is associated with increased mucosal T-cell infiltration and increased epithelial cell expression of CD74 and CD14

IF analysis of CD3 expression was used as an indicator of T-cell infiltration. A significant increase in the number of T cells recruited to both TLO and non-TLO mucosa, including intraepithelial lymphocytes, was observed in the proximal colons of CD_L3 mice compared to HCs (Figure 6a–c). The increase in T-cell infiltration in the distal colon of CD_L3 mice was less prominent, with no significant changes in the numbers of IELs observed. The plots and data distribution reflect the regional variability of T-cell numbers within the murine CD_L3 tissue and confirm the discontinuous inflammation characteristic of human CD.

(a) CD3^+^ T cells (green) in the colonic mucosa were analyzed by IF, and their numbers in the intraepithelial and lamina propria compartments of the proximal and distal colon were summarized and statistically analyzed by T test (b-c; **p < .01 ****p < .0001). (d) CD74 mRNA expression in the colons of humanized mice. (e) IF analysis of CD74 protein (in green; scale bars represent 100 μm). (f) CD14 expression was analyzed by IF in the proximal and distal colon. All scale bars are 50 μm. (g) Summary of the CD14 signal intensity (MFI-mean fluorescent intensity) in the surface and crypt colonic epithelial cells in the proximal and distal colon (T test; **p < .01 ****p < .0001). (H) CD44 mRNA expression in the colons of humanized mice.

The CD74 gene encodes the invariant chain (Ii), which is critical in the formation and transport of MHC class II peptide complexes for the generation of CD4^+^ T-cell responses and is upregulated in the epithelium of IBD patients^56–58^ where in response to inflammation, it contributes to tissue repair and mucosal healing by acting as the receptor for macrophage migration inhibitory factor (MIF)^58–61^ Fig. 6d depicts elevated *Cd74* mRNA expression in the proximal and distal segments of CD_L3 recipients. In the proximal colon of HC recipient mice, CD74-positive cells were limited to the immune cells in the lamina propria and the isolated lymphoid follicles (Figure 6e). The CD recipient proximal and distal colon showed a large increase in the amount of both CD74-positive lamina propria immune cells and increased CD74 expression in the surface epithelium. The observed changes tended to be less dramatic in the distal segment (Figure 6e).

CD14, the LPS-sensing coreceptor of TLR4, is expressed not only by myelomonocyte lineage cells but also by epithelial cells. Although the C-260T polymorphism in the CD14 gene has been associated with IBD in certain patient populations,^62^ it has also been shown to limit inflammatory responses by improving epithelial barrier function.^63^ In the proximal colon of mice colonized with HC microbiota, CD14 was detectable at the apical surface of the surface epithelial cells, with slightly less intense staining at the basolateral membranes and low expression in the crypts (Figure 6f). The same segment of the colon in CD_L3-colonized mice exhibited more intense CD14 staining of the surface epithelium, now also extending deeper into the crypts (Figure 6f). In the distal colon, CD14 expression did not differ between the HC and CD_L3 groups. The quantification summary shown in Figure 6g confirmed the interpretation of the IF images. RNA-seq analysis showed a more significant increase in the *Cd14* transcript in the proximal colon than in the distal colon of the CD_L3 group (Figure 6h). Interestingly, the change in *Cd14* expression in the distal colon of CD_L3 did not correspond with the protein abundance based on the IF data. Changes in mucosal expression of selected genes, including CD74 and CD14 were further validated by qRT-PCR (Fig. S5).

### Inflammation in CD microbiota recipients triggers colonic MMP7 expression

**Figure 7.**
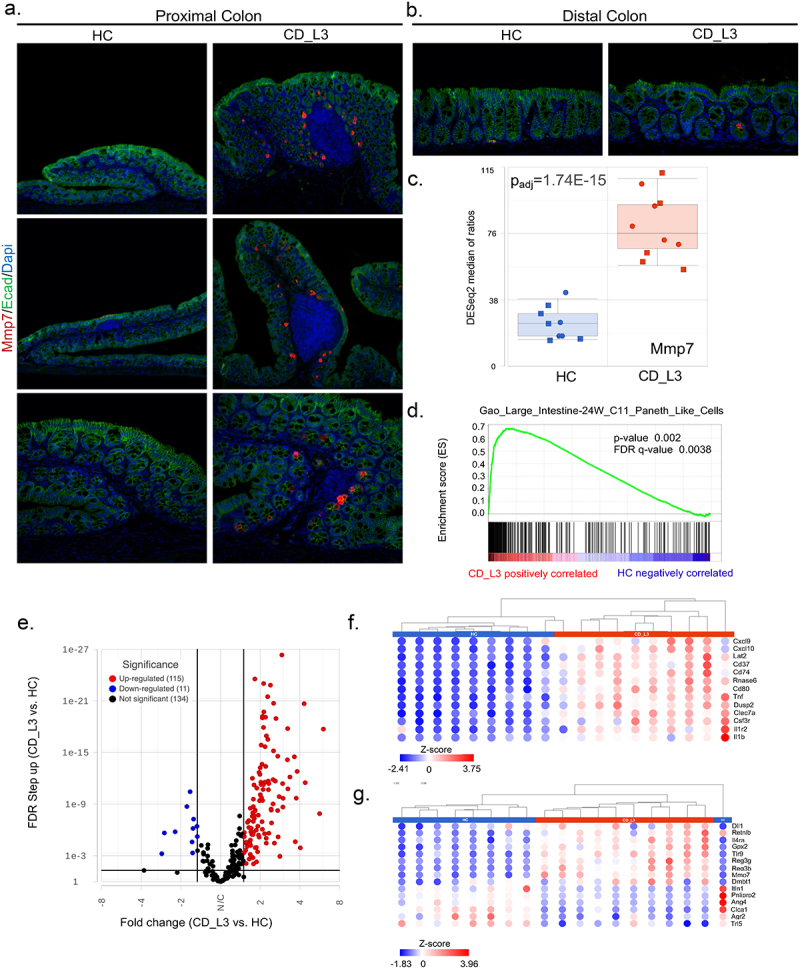
Paneth cell-like signature in the proximal colon of xGF mice humanized with HC and CD_L3 fecal microbiota.

The abnormal expression of MMP7, normally a Paneth cell-restricted matrix metallopeptidase, has been associated with colonic inflammation in both CD and UC as well as colorectal cancer^64–66^ GF mice colonized with HC microbiota did not show detectable MMP7 expression in the proximal or distal colon. However, in the proximal colon of CD_L3-associated mice, secretory cells in the immediate surrounding of the inflamed lymphoid follicles began to express MMP7 (Figure 7a). In the distal colon, the incidence of MMP7-expressing cells was very rare, with no apparent difference between experimental groups (Figure 7b). Increased MMP7 immunoreactivity in the proximal colon of CD_L3 recipient mice corresponded with increased *Mmp7* mRNA expression (Figure 7c). Although Paneth cell hyperplasia in the ascending colon and metaplasia in the descending colon have been well documented in IBD patients, they have not been described in rodent models of IBD. Our observations led us to speculate that secretory cells of the mouse proximal colon could be pushed toward differentiation to a Paneth-like cell phenotype as a response to persistent inflammation and the introduction of CD-associated microbes. Previous studies have demonstrated that both goblet and Paneth cells share a common secretory progenitor.^67^ To further investigate this, we performed gene set enrichment analysis (GSEA) with proximal colon RNA-seq data and identified a cell type signature consistent with the Paneth-like phenotype reported by Gao et al.^68^ in scRNAseq analysis of the developing human fetal large intestine (MSigDB systematic name M39160; *p* = .002, FDR q = 0.003) (Figure 7d). Of the associated transcripts, 188 had P_adj_ <0.05, and 127 had a fold change greater than 1.5 (Figure 7e). The heatmap in Figure 7f depicts 13 genes in this subset with a fold change ≥3 (Figure 7f). Figure 7g depicts genes that were also described as Paneth cell signature genes from the PanglaoDB database of murine and human scRNA projects.^69^

(a-b) MMP7 immunostaining in the proximal and distal colon of humanized mice. (c) Mmp7 mRNA expression in the proximal colon of humanized mice. (d) GSEA results indicating enrichment of transcripts associated with the Paneth-cell-like phenotype described in scRNAseq analysis of human immature small intestine.^68^ (e) Volcano plot of 260 Paneth cell-like gene markers from Gao et al.^68^ analyzed in the proximal colon of humanized mice. (f) Hierarchical clustering and heatmap of a selection of 13 genes from the Paneth cell-like signature with a fold change >3 in humanized mice. (g) Hierarchical clustering and heatmap of proximal colonic transcripts in humanized mice overlapping with Paneth cell signature genes from scRNA projects from PanglaoDB (consensus of scRNA sequencing data from mouse and human). All genes reported in the heatmap with P_adj_<0.05 (no fold-change adjustment).

## Discussion

Although the role of gut microbiota in the pathogenesis of IBD is beyond debate, the search for individual pathobionts and proinflammatory communities/enterotypes continues. Several studies have demonstrated that fecal transplantation of an IBD-associated dysbiotic community can confer susceptibility to colitis in xGF- or antibiotic-pretreated genetically susceptible or DSS-treated mice.^12–15^ Our data provide the first evidence that germ-free mice colonized with fecal microbiota from patients with Crohn’s ileocolitis are sufficient to induce mild to moderate chronic colonic inflammation in GF mice, in many respects reminiscent of the CD phenotype. This included a discontinued pattern of colitis, more proximal colonic localization, enlarged isolated lymphoid follicles and/or TLO neogenesis, as well as a transcriptomic pattern consistent with immune dysregulation in CD. To enable the establishment of a stable community while preventing long-term adaptive changes in the human microbiota of mice, we restricted our observations to the three-week post-colonization period. Consequently, we were unable to determine conclusively whether the observed inflammatory changes were transient, occurring during the recovery phase in mice, or if they were indicative of a progressive disease manifesting in its early stages. Given that the mice utilized lacked genetic susceptibility to inflammatory bowel disease (IBD), it appears more probable that the former scenario holds true.

Previous studies that have demonstrated the exacerbation of murine colitis by FMT from IBD patients did not report the efficiency of engraftment of the complex human fecal communities in mice.^12–15^ Metagenomic compositional analysis in our study suggested only 27.9% engraftment efficiency with FMT from healthy patients and an even lower, 8.6% efficiency with CD microbiota. Low engraftment may be explained by host species differences and lack of genetic IBD susceptibility traits in the murine recipients. Indeed, only 15% of bacterial species in the distal gut microbiota were shown to be shared between humans and mice.^70^ Fecal sample collection, storage, FMT preparation, and the associated exposure to ambient oxygen could further confound colonization by partial elimination of obligate anaerobes, especially those unable to form spores. The observed much lower engraftment of the CD community compared to the HC community may be explained by the generally lower microbial diversity of these samples, but it is also plausible that the CD microbiome is more unstable or prone to disruption and, as a result, generally more difficult to transfer across species.

Characterization of microbiota from ileal and fecal samples of recipient xGF mice reflected the differences in community diversity among the human specimens. Specifically, fecal contents from CD_L3 recipients had significantly lower richness, Shannon, and Simpson indices of alpha diversity compared to the HC recipients. However, in the ileal contents, only richness reflected a significant difference in alpha diversity between the two groups. Although beta diversity analysis showed altered ileal microbiota composition between CD_L3 and HC recipients, this did not translate to morphological or transcriptional changes consistent with ileitis, which suggests that fecal microbiota is not capable of promoting inflammation in this segment and/or is not able to recapitulate the human ileal community in mice.

Among the bacteria consistently overrepresented in CD_L3 patient samples and recipient mice were *Ruminococcus gnavus, Erysipelatoclostridium ramosum, Faecalimonas umbilicate, Blautia hominis*, *Clostridium butyricum*, and *C. paraputrificum*, with a longer list of 48 ASVs with consistently reduced abundance. Interestingly, the mucin consumer *R. gnavus*, known for its proinflammatory association with Crohn’s disease and experimental colitis^48,71^ was dramatically more elevated between mouse recipients of CD_L3 microbiota compared to HC recipients than in the source donor samples. We previously reported that mice lacking Paneth cell lysozyme had significantly elevated *R. gnavus* colonization.^48^ These results suggest that the limited IBD-derived microbial community and alteration of the host luminal environment in mice may have established conditions favorable to *R. gnavus* growth and dominance.

Although no pathogenic bacteria were identified among ASVs concordant between human inoculum and mouse fecal samples, we identified three bacterial species in GF recipients that were below detection levels in human donor samples: *Eisenbergiella massiliensis, Clostridium_sp_7_2_43FAA*, and *Clostridioides difficile*. All three have been described in human fecal samples^72–74^ but only *C. difficile* gained notoriety as an infectious cause of pseudomembranous colitis^75^ and a pathogen that complicates the outcomes in IBD patients and in murine models.^76,77^ The unexpected expansion of toxigenic tcdA- and tcdB-positive *C. difficile* in CD_L3 recipient mice is a novel and fascinating observation, as it describes a reduced human microbial community highly capable of promoting its growth. This may be of high relevance to the understanding of the key players among the healthy gut microbial community that limit *C. difficile* growth and the associated host response, as recently shown in an elegant study by Nagao-Kitamoto et al.^78^ and contribute to the growing efforts to identify bacteria promoting or restricting pathogen growth in humanized xGF mice^79–82^ It is difficult, however, to establish whether the pathogenic response in our mice could be solely attributed to *C. difficile* growth. Although its relative abundance reached ca. 4% in CD_L3 FMT recipients, in human CDI patients and in a mouse CDI model, fecal *C. difficile* burden can vary dramatically and does not correlate with any measure of illness or outcome.^83–85^ Moreover, the histological changes observed in the CD_L3 microbiome recipients were not consistent with those reported by others in GF or antibiotic-treated mice, typically with more fulminant disease with diarrhea and the associated high mortality. Because there is no selective treatment for *C. difficile*, we could not determine the role of *C. difficile* in our model, and any antibiotic treatment would likely further destabilize the transplanted community and make the results uninterpretable.

CD_L3 FMT was associated with increased epithelial expression of CD14, a CD susceptibility gene and LPS coreceptor, as well as CD74, normally limited to the mucosal immune compartment. CD74, a major histocompatibility complex class II chaperone and a receptor for the cytokine macrophage migration inhibitory factor (MIF), is increased in IBD^56,57^ and its epithelial cell expression was recently described to contribute to mucosal restitution.^58^ It is possible that increased CD74 expression in our model limited the inflammatory response and contributed to the relatively mild tissue damage.

MMP7 is one of the markers of small intestinal Paneth cells, where it is responsible for activating α-defensins.^86^ Although normally absent in a healthy colon, elevated colonic expression of MMP7 has been previously shown in the endothelial cells and infiltrating leukocytes of the inflamed colonic mucosa of IBD patients,^66^ and it has been associated with epithelial barrier dysfunction.^87^ However, our IF analysis showed increased but sparse MMP7 immunostaining within the proximal and, to a much lesser extent, distal colonic crypt epithelium, which, we speculate, could represent the precursors leading to Paneth cell metaplasia. Indeed, one of the novel observations in CD_L3-associated xGF mice was a transcriptomic signature of the Paneth cell-like profile in the proximal colon. Paneth cell hyperplasia in the ascending colon and metaplasia in the descending colon are typical features in IBD patients but have not been described in animal models of IBD. It is plausible that the development of the Paneth-like secretory lineage in the inflamed colon is unique to humans due to the associated microbial cues that conventional mice are lacking. Our results point to only a partial Paneth-like signature (e.g., no increase in lysozyme expression), suggesting that additional elements, either host-derived or microbial in origin, were still missing. Future studies will need to address this fascinating concept in more detail.

In summary, our study provides the first evidence that the transfer of a dysbiotic community from CD patients can lead to spontaneous inflammatory changes in the colon of xGF mice. Although there are limitations to the use of humanizing mice with human fecal microbiota, including a limited engraftment of the complex community, it may have also been a fortuitus finding that will allow the field to further narrow down the microbial composition capable of promoting the growth and relative expansion of pathobiont and pathogenic bacteria, which can promote epithelial and immune dysfunction and lead to chronic intestinal inflammation.

## Supplementary Material

Supplemental Material

## Data Availability

All raw data from microbiome analysis and RNAseq transcriptome profiling have been submitted to the NCBI Sequence Read Archive (SRA) database under BioProject PRJNA981221 and BioProject PRJNA980747, respectively.
