# Supplementary material for: Transplant of microbiota from Crohn’s disease patients to germ-free mice results in colitis
Source: Gut Microbes. 2024 Mar 27;16(1):2333483. doi: 10.1080/19490976.2024.2333483 (PMC10978031; doi:10.1080/19490976.2024.2333483)
Supplement: Supplemental Material [file KGMI_A_2333483_SM3348.zip › Supplemental figure legends_clean.docx]

**SUPPLEMENTAL FIGURE LEGENDS**

**Figure S1. Visual representation of the relative genus abundance in** **fecal samples from HC, CD_L1, and CD_L3 patients.** Only genera with relative abundance of > 0.1% are shown.

**Figure S2. Real-time qPCR analysis of C. difficile 16S and *tcdA* and *tcdB* genes in the fecal samples of mice humanized with HC and CD_L3 microbiota.** qPCR was run for 45 cycles and Cq values for individual samples and genes is shown. Cq of 45 was arbitrarily assigned to samples where genes were not amplified. Pan-bacterial 16S primer/probe set (total 16S) was used as loading control.

**Figure S3. Metagenomic identification of C. difficile in CD_L3 microbiota recipient mice. (A)** Coverage of K100365 per sample. (**B**) The pathogenicity loci within the analyzed contigs. In the case of the ♀CD_L3 locus, it was detected on the reverse DNA strand. (**C**) Top 10 results of BLAST search for the extracted contigs.

**Figure S4. Ileum is not significantly affected in mice humanized with CD_L3 microbiota.** Ileal histology (**A**) and immunostaining for Paneth cell markers, LYZ1, MMP7, and Mptx2 (**B-D**) in the ileum of humanized mice. All scale bars represent 100 μm. (**E**) Principal component analysis (PCA) of ileal transcriptome in HC and CD_L3 humanized mice. (**F**) Lyz1, Mmp7, and Mptx2 mRNA expression in the ileum of humanized mice. (**G**) Volcano plot demonstrating limited changes in the ileal transcriptome of humanized mice. (**H**) Histological inflammation scores indicate lack of inflammatory changes in the ileum of HC and CD_L3 humanized mice.

**Figure S5.** **Validation of selected genes in the proximal and distal colon of** **mice humanized with healthy control or CD_L3 microbiota.** qRT-PCR was used to analyze the expression of selected genes (TNFα, CD74, CD14, Cxcl9, Cxcl10, and CIITA) with TATA box binding protein (TBP) used as an internal control. Normal distribution of data was confirmed using Shapiro-Wilk test and expression values compared between HC and CD_L3 samples using Student t-test. P values are indicated for each data sets.

**Figure S6. The colonic transcriptomic profile of CD_L3 recipients shows an enrichment in the IBD pathway**. KEGG IBD signaling pathway (redrawn for resolution and clarity) with genes differentially expressed in the colon of HC and CD_L3 recipients. Genes upregulated in CD_L3 are highlighted in red, and those downregulated are highlighted in green.
