# Supplementary material for: Transplant of microbiota from Crohn’s disease patients to germ-free mice results in colitis
Source: Gut Microbes. 2024 Mar 27;16(1):2333483. doi: 10.1080/19490976.2024.2333483 (PMC10978031; doi:10.1080/19490976.2024.2333483)
Supplement: Supplemental Material [file KGMI_A_2333483_SM3348.zip › Supplemental Table S1.docx]

**Supplemental Table S1.** Patient characteristics.

|  | *Healthy Controls (HC)* |  |
| --- | --- | --- |
|  | N (Male:Female) | 29 (7:22) |
|  | Median age (range) | 39 (19-69) |
|  |  |  |
|  | ***Crohn's disease (CD)*** |  |
|  | N (Male:Female) | 35 (22:13) |
|  | Median age (range) | 36 (18-63) |
| Age of onset | A1 (<16 yrs) (N) | 6 |
|  | A2 (17-40 yrs) (N) | 26 |
|  | A3 (>40 yrs) (N) | 3 |
| CD location | L1 (ileal) | 6 |
|  | L2 (colonic) | 6 |
|  | L3 (ileocolonic) | 23 |
|  | L4 (isolated upper) | 0 |
| Disease behavior | B1 (non-sticturing, non-penetrating) | 18 |
|  | B2 (stricturing) | 8 |
|  | B3 (penetrating) | 8 |
|  | B3p (penetrating with perianal disease) | 1 |
