# Supplementary material for: Transplant of microbiota from Crohn’s disease patients to germ-free mice results in colitis
Source: Gut Microbes. 2024 Mar 27;16(1):2333483. doi: 10.1080/19490976.2024.2333483 (PMC10978031; doi:10.1080/19490976.2024.2333483)
Supplement: Supplemental Material [file KGMI_A_2333483_SM3348.zip › Supplemental Table S2.docx]

**Supplemental Table S2**. Primers (forward and reverse; -F, -R) and TaqMan probes (-P) used in toxigenic *C. difficile* screening by qPCR.

| **Primer/probe name** | **Sequence** | **Final concentration** |
| --- | --- | --- |
| CDiff16sRNA-F | GCAAGTTGAGCGATTTACTTCGGT | 900nM |
| CDiff 16sRNA-R | GTACTGGCTCACCTTTGATATTYAAGAG | 900nM |
| CDiff 16sRNA-P | **FAM**-TGCCTCTCAAATATATTATCCCGTATTAG-**TAMRA** | 250nM |
| tcdA-F | CAGTCGGATTGCAAGTAATTGACAAT | 900nM |
| tcdA-R | ATGAGTATCTACTACCATTAACAGTCTGC | 900nM |
| tcdA-P | **FAM**-TTGAGATGATAGCAGTGTCAGGATTG-**TAMRA** | 250nM |
| tcdB-F | TACAAACAGGTGTATTTAGTACAGAAGATGGA | 900nM |
| tcdB-R | CACCTATTTGATTTAGMCCTTTAAAAGC | 900nM |
| tcdB-P | **FAM**-TTTKCCAGTAAAATCAATTGCTTC-**TAMRA** | 250nM |
