# Supplementary material for: Transplant of microbiota from Crohn’s disease patients to germ-free mice results in colitis
Source: Gut Microbes. 2024 Mar 27;16(1):2333483. doi: 10.1080/19490976.2024.2333483 (PMC10978031; doi:10.1080/19490976.2024.2333483)
Supplement: Supplemental Material [file KGMI_A_2333483_SM3348.zip › Supplemental Table S3.docx]

**Supplemental Table S2.** Relative abundance of the 72 species of bacteria transferred to GF mice from healthy control (HC) or Crohn’s disease (CD_L3) pooled patient samples identified by shotgun metagenomics. Top part of the table lists species increased or decreased in relative abundance in CD_L3 donors and in both male and female recipient mice (congruent across sex). Middle part groups species generally with the same directionality of change, but not congruent across mouse sex. Bottom part lists species generally not congruent between donor and recipient samples.
