# Supplementary figures and images for: Transplant of microbiota from Crohn’s disease patients to germ-free mice results in colitis
Source: Gut Microbes. 2024 Mar 27;16(1):2333483. doi: 10.1080/19490976.2024.2333483 (PMC10978031; doi:10.1080/19490976.2024.2333483)

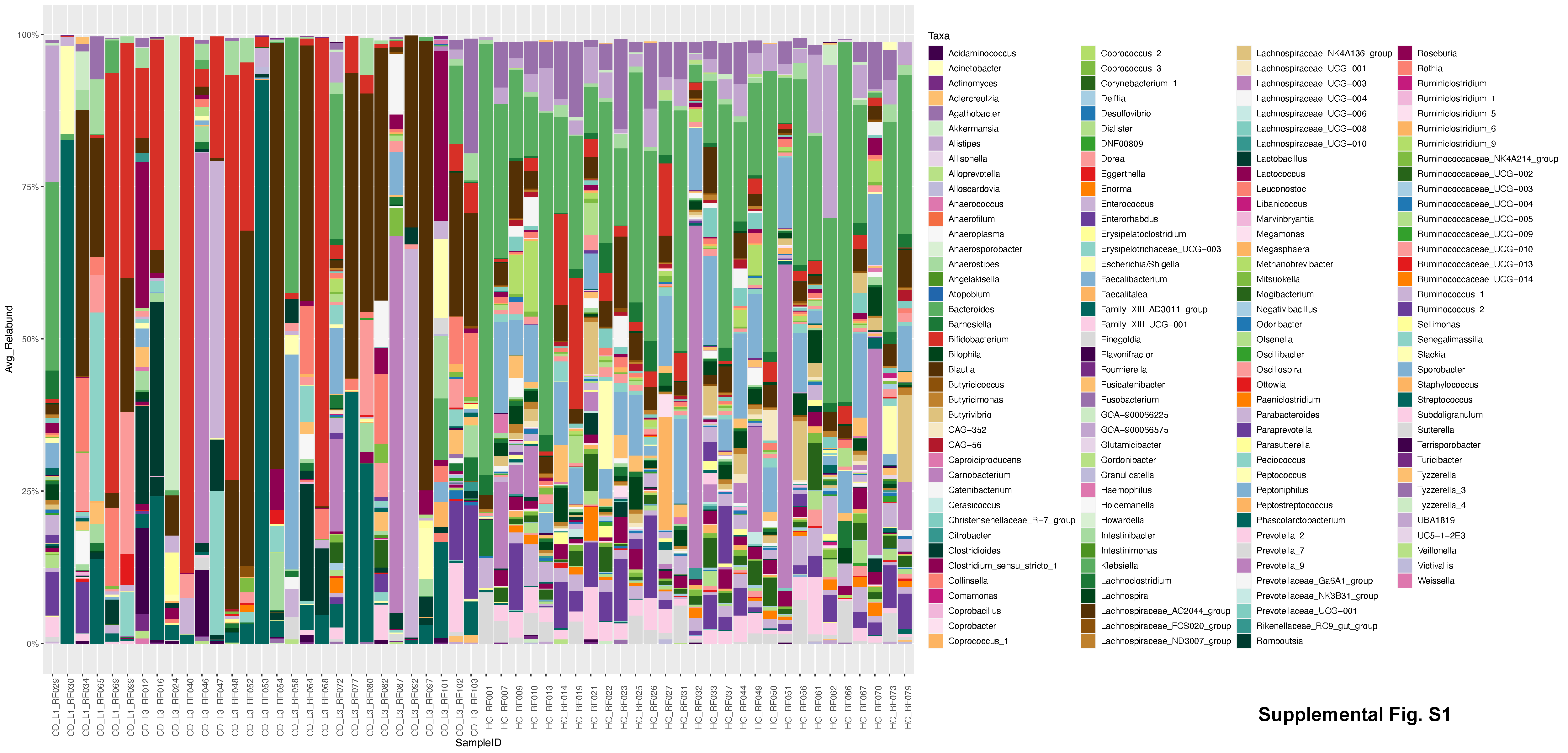

Supplement: Supplemental Material [file KGMI_A_2333483_SM3348.zip › Fig S1.tiff]

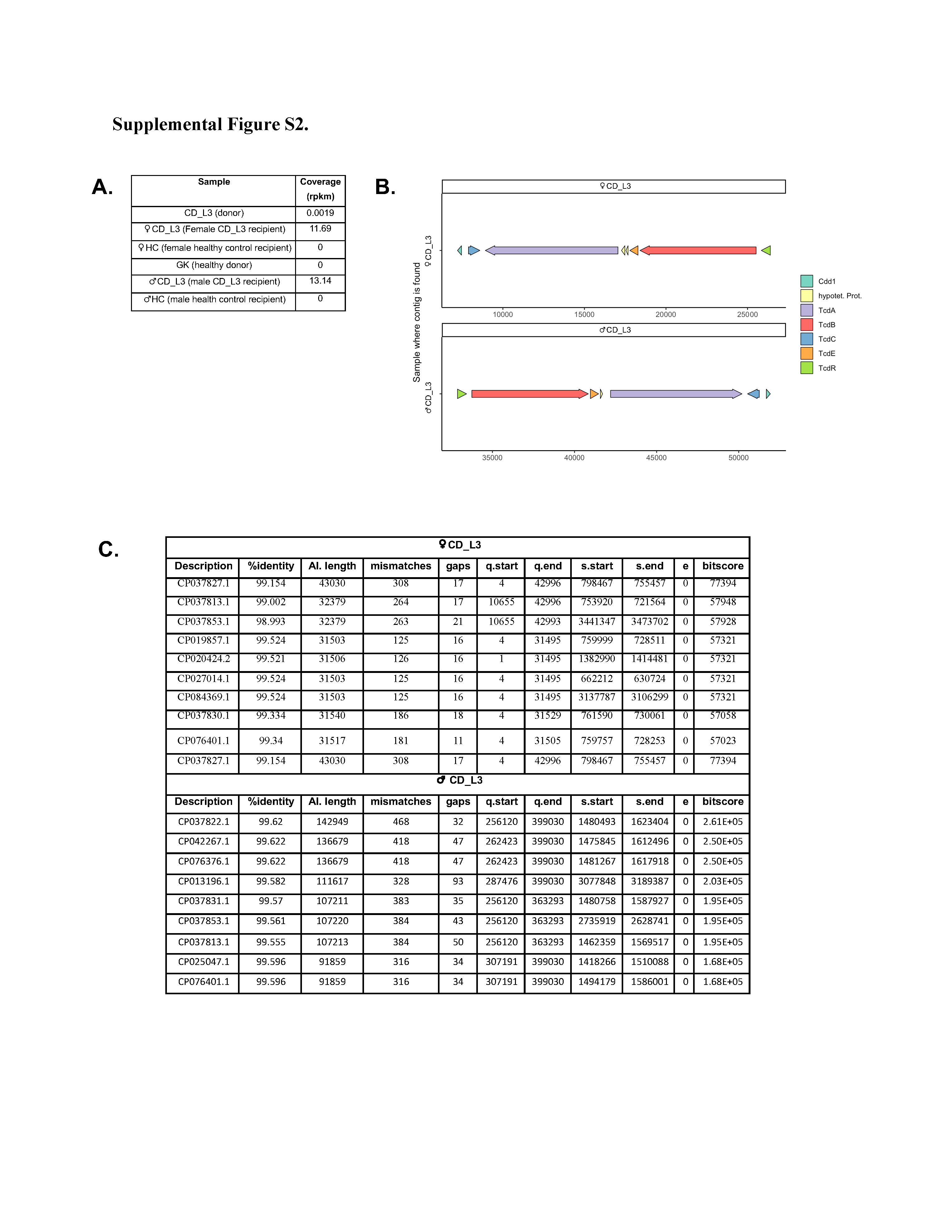

Supplement: Supplemental Material [file KGMI_A_2333483_SM3348.zip › Fig S2.tiff]

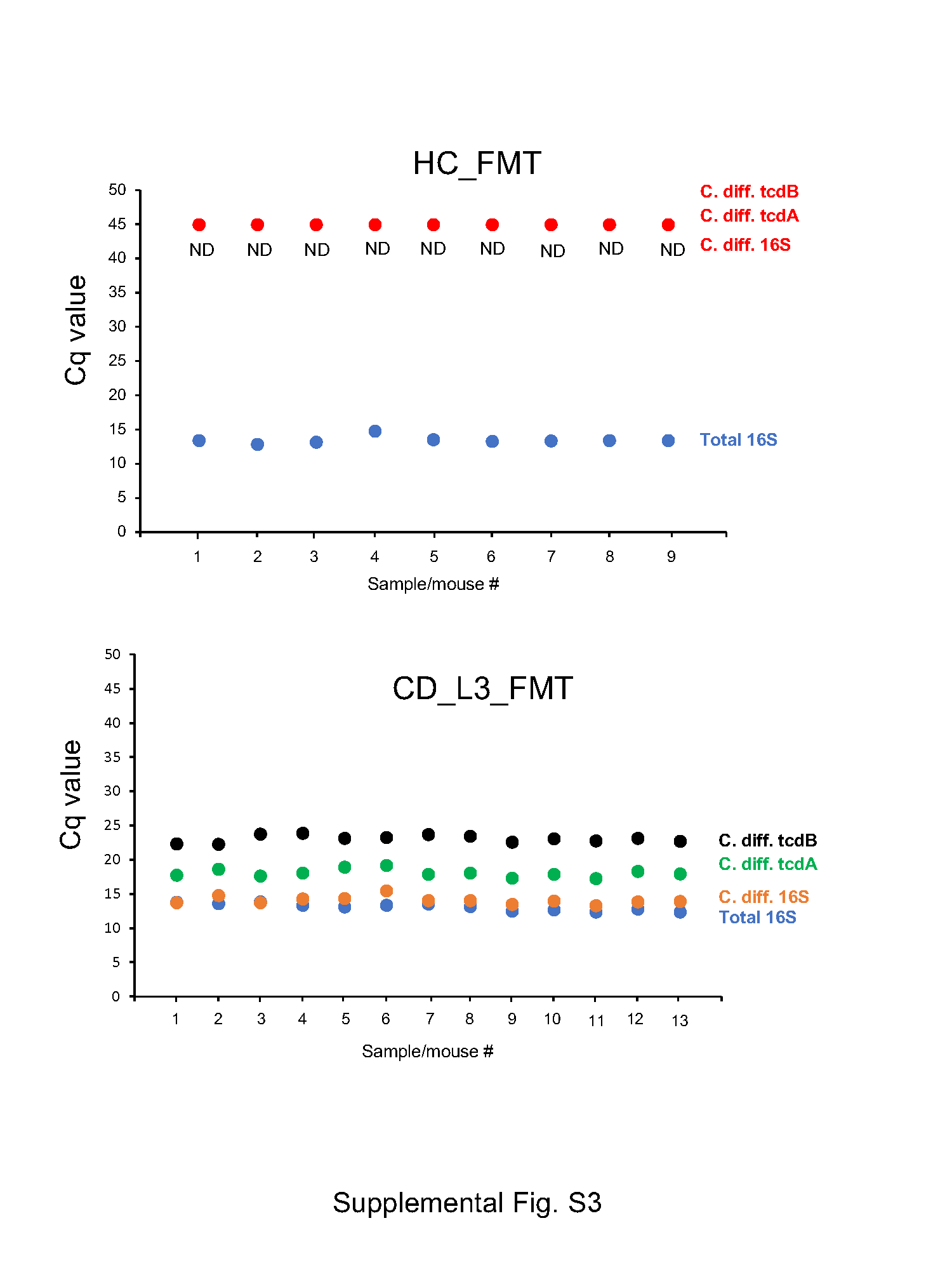

Supplement: Supplemental Material [file KGMI_A_2333483_SM3348.zip › Fig S3.tiff]

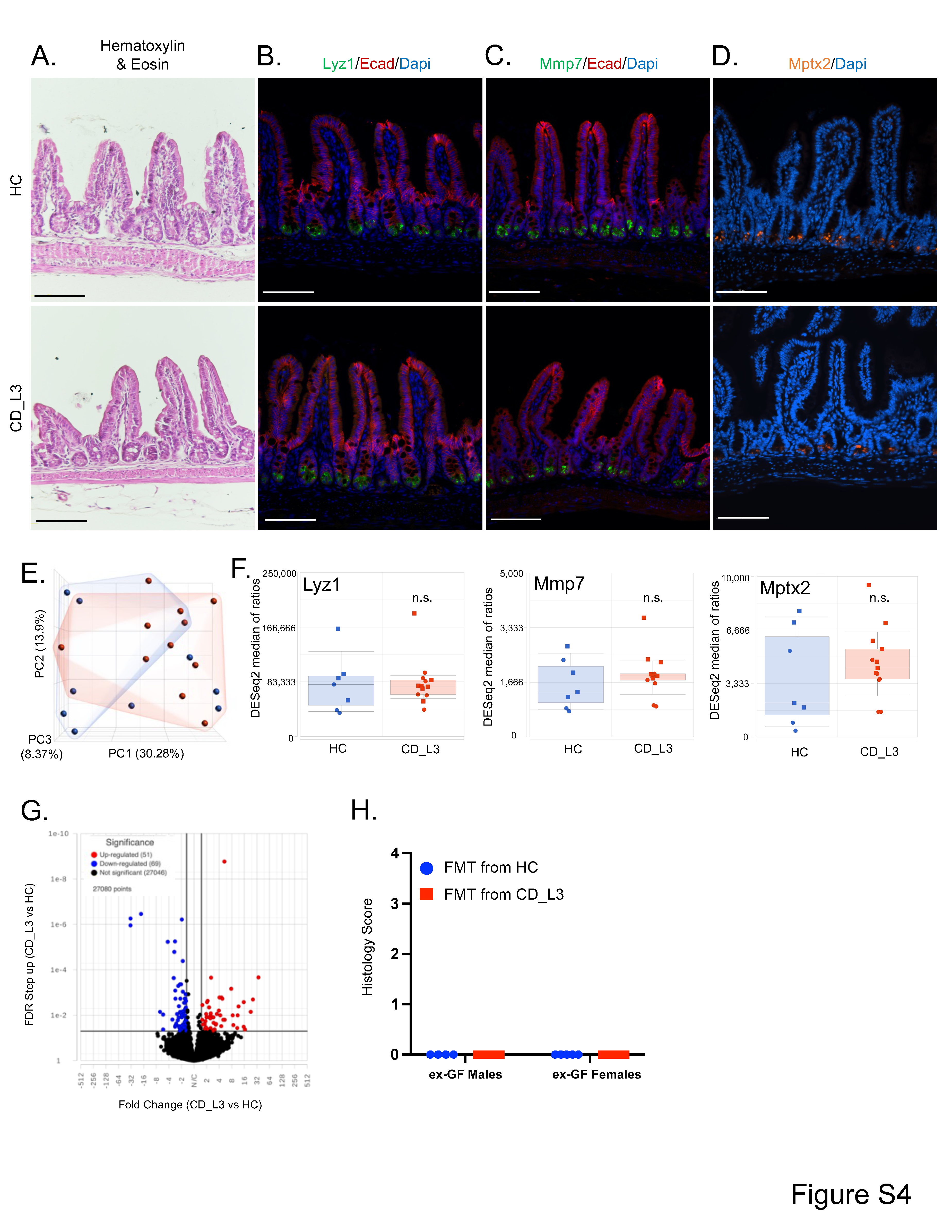

Supplement: Supplemental Material [file KGMI_A_2333483_SM3348.zip › Fig S4.tiff]

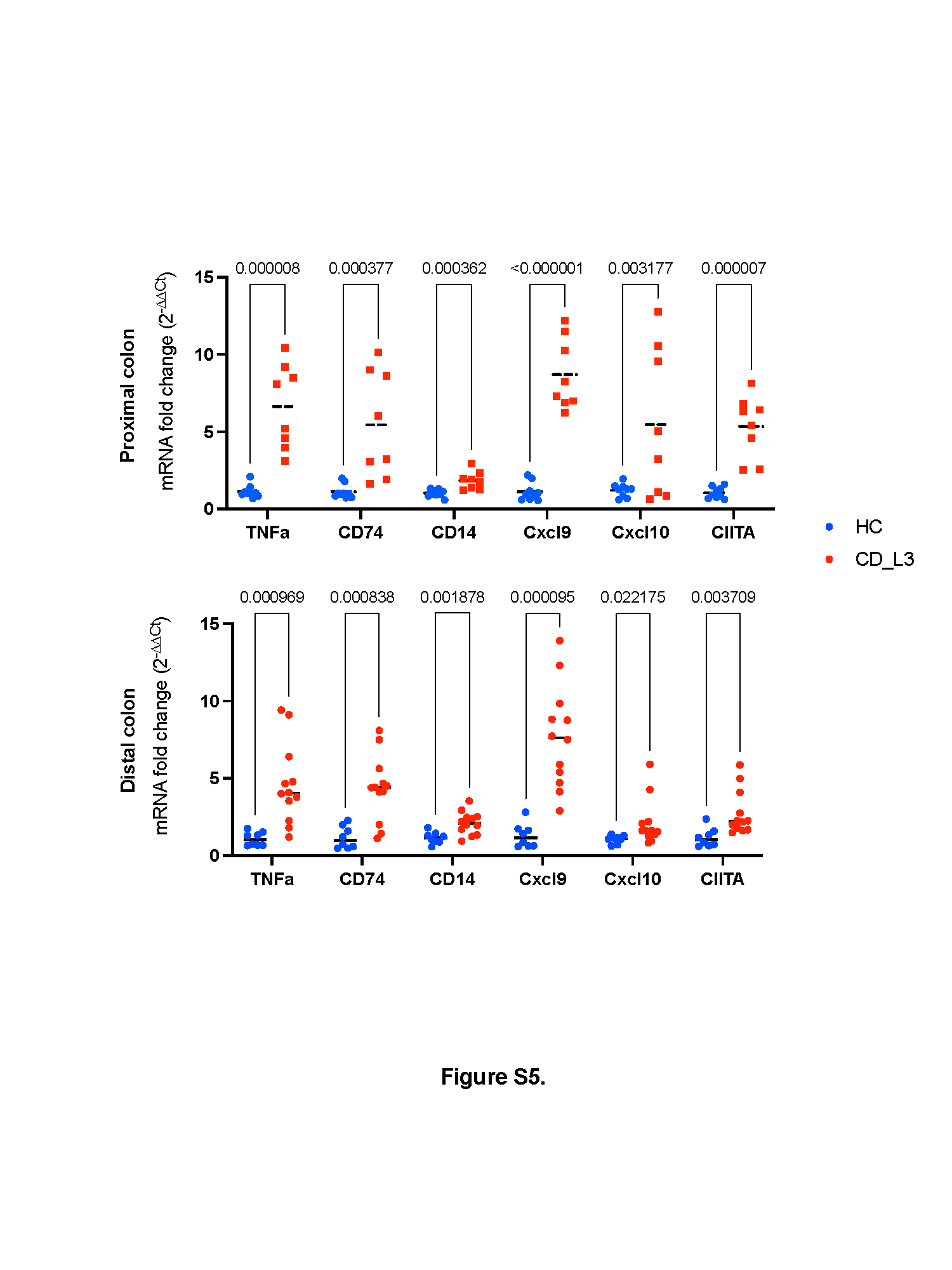

Supplement: Supplemental Material [file KGMI_A_2333483_SM3348.zip › Fig S5.tiff]

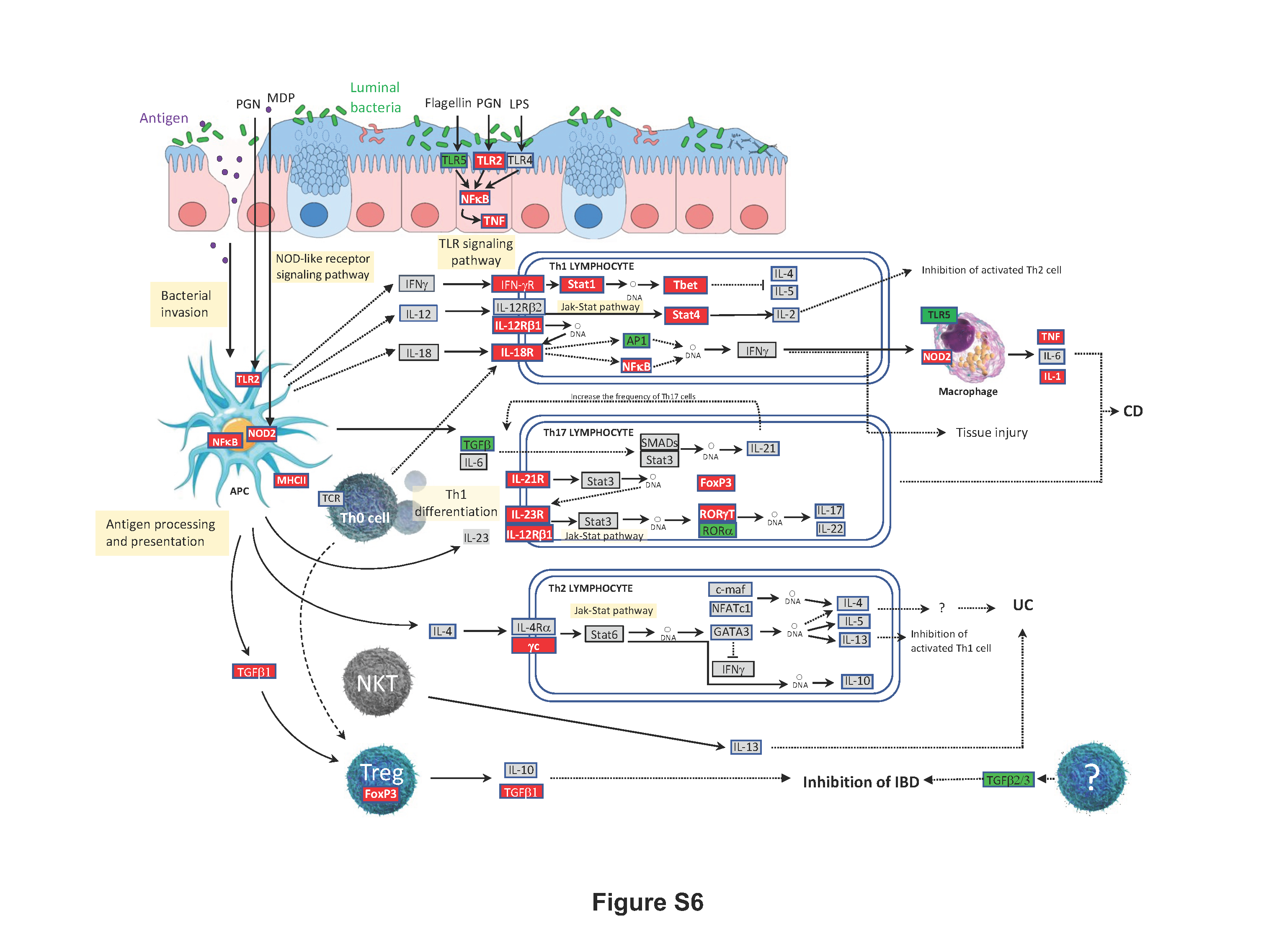

Supplement: Supplemental Material [file KGMI_A_2333483_SM3348.zip › Fig S6.tiff]
